# Factors contributing to helminth prevalence after repeated mass administration of medicines in Anambra State, Nigeria

**DOI:** 10.1038/s41598-024-83063-6

**Published:** 2025-01-29

**Authors:** Ogechukwu B. Aribodor, Christopher Okaka, Sammy Sam-Wobo, Annick Bikoumou, Emmanuel Obikwelu

**Affiliations:** 1 Department of Zoology, Nnamdi Azikiwe University, Awka, Nigeria; 2 Department of Animal and Environmental Biology, University of Benin, Benin City, Nigeria; 3 Department of Pure and Applied Zoology, Federal University of Agriculture, Abeokuta, Nigeria; 4 WHO Africa Regional Office, Universal Health Coverage, Communicable & Non-Communicable Diseases (UHC/CND) Cluster, Brazzaville, Congo; 5 Neglected Tropical Diseases Unit, Anambra State Ministry of Health, Awka, Nigeria

**Keywords:** Community engagement, Mass Administration of Medicines, Schistosomiasis, Soil-transmitted helminthiasis, Sustainable intervention, Epidemiology, Outcomes research

## Abstract

Over the past decade, Mass Administration of Medicines (MAM) has been a key strategy for controlling schistosomiasis and soil-transmitted helminthiasis (STHs) in Anambra State, Nigeria. This longitudinal study, conducted from 2017 to 2019, evaluated the impact of interventions for controlling schistosomiasis (SCH) and STHs in recipient communities. A total of 1,046 pupils aged 5 to 16 years were enrolled, with Kato-Katz and urine filtration methods used for faecal and urine sample analysis. A structured questionnaire was administered to 243 people to assess the contextual factors. At baseline, prevalence was 8% (82/1046), with *A. lumbricoides* (7.0%), *T. trichiura* (1.0%), Hookworm (0.1%), and *S. haematobium* (0.5%) observed. Co-infection was 1%. At follow-up, prevalence decreased to 6% (65/1046), with *A. lumbricoides* (2.0%), *T. trichiura* (2.2%), and *S. haematobium* (2%) observed, and co-infection was 0.2%. Infection levels varied by location (*p* > 0.05), with socio-economic status and inadequate WASH (Water, Sanitation, and Hygiene) infrastructure contributing to transmission risk. Most respondents (87%) earned less than $50 per month, and 39% practiced open defecation. The persistence of open defecation highlights critical gaps in WASH that undermine sustainable Neglected Tropical Diseases (NTD) control. Addressing cultural and economic challenges, alongside improving WASH infrastructure, is essential to sustain MAM’s impact.

## Introduction

Helminthiasis among pupils is a major impediment to their well-being and development. Over the past decade, Mass Administration of Medicines (MAM) has been a cornerstone in controlling schistosomiasis and soil-transmitted helminthiasis (STH) in Anambra State, Nigeria^1–3^. This longitudinal study, conducted from 2017 to 2019, evaluated the implementation and impact of these interventions in the affected communities, aiming to provide actionable insights for sustainable disease control and elimination. The study provides actionable insights for sustainable disease control and elimination, particularly in the context of ongoing global health challenges.

Schistosomiasis and soil-transmitted helminthiasis are public health concerns and significant impediments to socio-economic development^4^. Intestinal helminthiasis among pupils, for instance, is a major barrier to their well-being and cognitive development, leading to long-term economic consequences due to reduced educational attainment and productivity^5,6^. Schistosomiasis, with its debilitating effects, hinders physical and cognitive growth, thereby perpetuating cycles of poverty and economic disparity. Addressing these diseases is crucial for achieving broader health and economic goals, as emphasized in the World Health Organization (WHO) Neglected Tropical Diseases (NTD) Road Map 2021–2030^6^.

The World Health Organization (WHO) Neglected Tropical Diseases (NTD) Road Map 2021–2030 heralds a paradigm shift from disease-specific interventions to integrated, cross-cutting approaches within existing health systems. This transition is essential for fostering the community sustainability of MDA programs^6^. The road map’s emphasis on mainstreaming interventions into health systems highlights the critical role of community engagement in achieving long-term success and sustainability. Strengthening program delivery by incorporating community perceptions and opinions is crucial for promoting equitable, person-centered service delivery^7^.

Active community participation is indispensable for the success of any control program, including MDA. Studies underscore that misunderstandings about an intervention or skepticism regarding its benefits can lead to low participation rates. Conversely, when communities understand and agree with the goals and methods of an intervention, their involvement and support increase significantly^8–10^. Therefore, community engagement is a pivotal, yet frequently overlooked, aspect of control and elimination programs. Effective community empowerment through knowledge, engagement, collaboration, and leadership, combined with improved infrastructure, can bring about sustainable changes that reduce the risk of acquiring schistosomiasis and STH^6,7^.

The COVID-19 pandemic has underscored the importance of community resilience and sustainable health interventions. The timing of this study, completed just weeks before the lockdown, highlights the critical need for robust community engagement strategies to ensure continuity and effectiveness of health programs in the face of global health crises. The pandemic’s impact on funding for NTD programs, particularly the significant cuts by the UK Foreign and Commonwealth Development Office (FCDO), has further emphasized the need for alternative funding mechanisms^11^. Public engagement and crowdfunding are now critical to ensuring the continuity of research and intervention programs for neglected tropical diseases^12^. Thus, community involvement is vital not only for program implementation but also for sustaining financial support in the long term^12,13^.

The WHO Regional Office for Africa’s Accelerated NTD Mapping Project, initiated in 2013, has highlighted the importance of epidemiological mapping for effective intervention planning^14,15^. Mapping provides essential data on disease distribution and endemicity, which is critical for targeting interventions and ensuring efficient resource utilization. Accurate mapping helps avoid wastage and ensures that treatment reaches the populations most in need^6^.

In Nigeria, schistosomiasis and STHs are major public health concerns, with mapping showing that 583 out of 774 local government areas (LGAs) are endemic for schistosomiasis^1^. Despite substantial progress in treatment coverage, challenges remain in ensuring the sustainability and effectiveness of MDA programs. The mapping of Soil-Transmitted Helminths (STH) and schistosomiasis in Anambra State was carried out in a phased manner between 2011 and 2013. The mapping of STH began in 2012 across Awka North, Ayamelum, Ekwusigo, Ihiala, and Orumba South, and continued in 2013 to include Aguata, Awka South, Dunukofia, Njikoka, Nnewi North, Nnewi South, and Oyi Local Government Areas (LGAs). Schistosomiasis mapping commenced earlier, in 2011, with Anambra West LGA, expanded in 2012 to Idemili North, Ogbaru, Onitsha South, and Orumba South, and extended further in 2013 to Awka South, Dunukofia, Njikoka, Nnewi North, Nnewi South, and Orumba South^3^. These mapping efforts identified endemic areas, leading to school-based chemotherapy with Albendazole and Praziquantel^1–3^. However, some communities were excluded from the intervention because they were classified as non-endemic^1,3^. No subsequent comprehensive mapping has been conducted in the state since then, raising concerns about potential misclassification of communities and restricted access to preventive chemotherapy (PC) programs. This oversight could mean that some populations at risk may have been excluded from necessary treatments. However, achieving sustainable disease control requires not just periodic treatment but also comprehensive measures to address the environmental and socio-economic factors that contribute to disease transmission.

Mass administration of medicines alone is unlikely to break the transmission cycle of schistosomiasis and STHs without complementary environmental measures such as improved sanitation and access to clean water. Health education is also critical for encouraging behaviours that reduce infection risk. Modelling studies suggest that community-wide MAM delivery, combined with high coverage and compliance, can potentially interrupt disease transmission. Nevertheless, achieving such outcomes requires overcoming significant challenges, including the risk of anthelmintic resistance and the premature dismantling of NTD programs due to funding constraints^6,11^.

The WHO NTD Road Map’s deadline of 2030 is now less than six years away, making it crucial to accelerate efforts to achieve the outlined targets. This urgency underscores the relevance of this study, which addresses the pressing need for a systematic evaluation of ongoing MAM programs in Anambra State. The limited evidence on the impact of ongoing Mass Administration of Medicines (MAM) programs in Anambra State underscores the need for surveillance, monitoring, and evaluation efforts. This knowledge gap not only hinders our understanding of the initiatives’ effectiveness but may also inadvertently deprive certain populations and communities of essential treatments.

This study aims to address the pressing need for a systematic evaluation of the ongoing MAM programs in Anambra State. It assesses the current prevalence of schistosomiasis and STHs, evaluates the efficacy of drug administration programs, and identifies socio-economic and behavioural factors influencing disease transmission. By understanding these factors, the study seeks to develop strategies for complete community involvement in managing and sustaining drug administration programs. Ultimately, this research aims to contribute to the control and possible elimination of schistosomiasis and soil-transmitted helminthiasis in the region, while also supporting the global effort to eliminate neglected tropical diseases, promote health equity, and improve access to quality healthcare.

## Methods

### Study area

Anambra State is located in the south eastern part of Nigeria with a landmass of about 4,844 km^2^ (1,870 sq. m) and a population of 4,177,828. It has geographical coordinates of 6°20′0′ North 7°0′0′ east. Anambra is the eighth-most populated state in the Federal Republic of Nigeria and the second-most densely populated state after Lagos State^16^. It has tropical rainforest vegetation, with two distinct seasons in the year, rainy (April to October) and the dry season (November to March). The area is rich in natural gas, crude oil, bauxite, and ceramic. It has an almost 100% arable soil. The state has many other resources in terms of agriculturally based activities such as fisheries and farming, as well as land cultivated for pasturing and animal husbandry. Open defecation practices are also very common as there is limited availability of functional toilet facilities and safe water supply in most of the towns within the state. Power supply is almost non-existent in these communities. Poorly equipped health centres, absence of standard laboratories and lack of trained health personnel indicate that public health outreaches in these communities are rare.

**Fig. 1:**
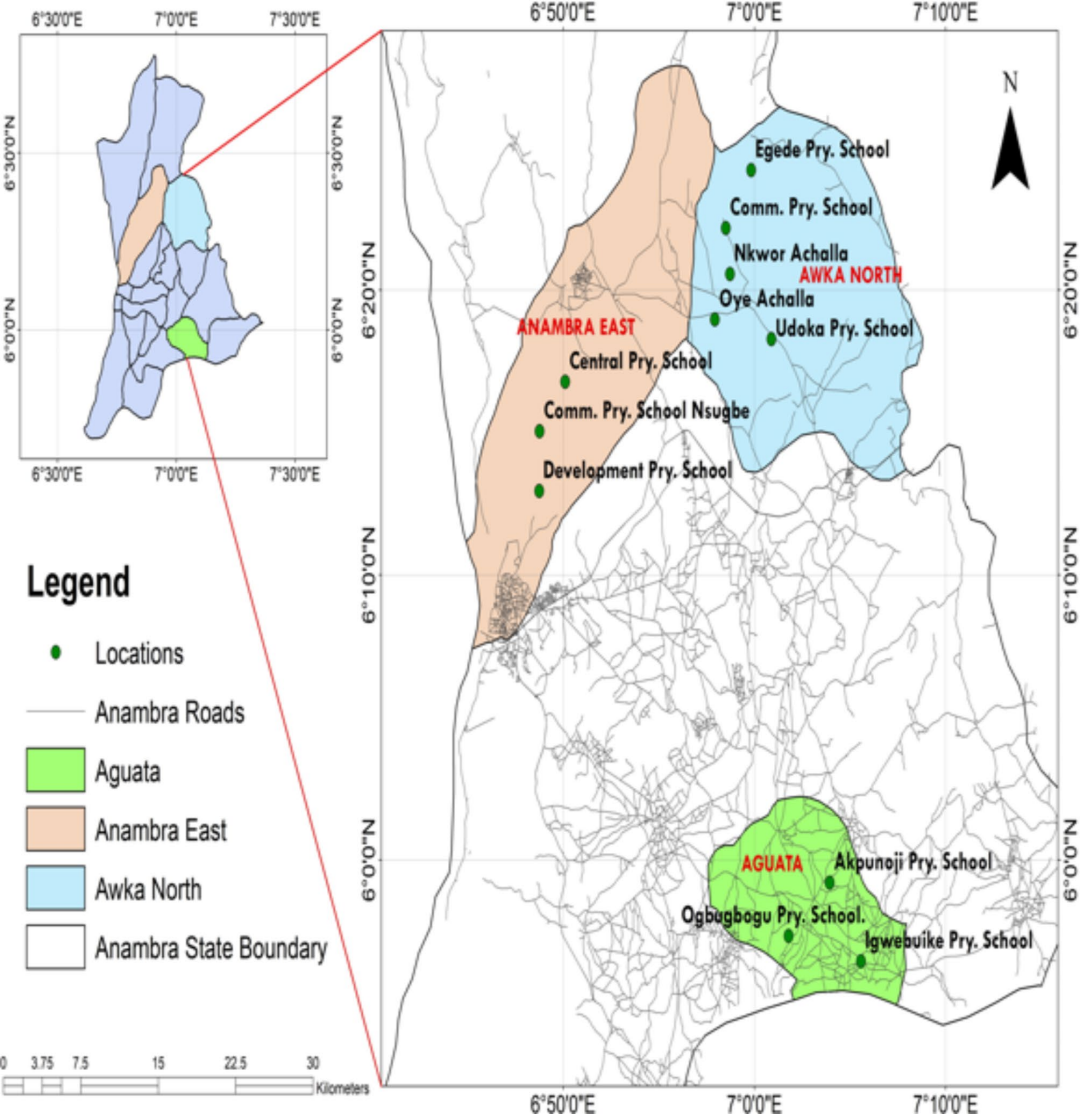
Map of Anambra State Showing the study locations within the Local Government Areas chosen for the Study (Source: Geography Information System Laboratory, Department of Estate Survey and Geoinformatics, Nnamdi Azikiwe University, 2019).

The study was carried out in three communities selected by the Ballot method^17^ namely; Achalla in Awka North Local Government Area (LGA), Ezinifite in Aguata LGA and Nsugbe in Anambra East LGA and covering the three senatorial zones of Anambra State selected (Fig. 1).

### Study design

The longitudinal study was conducted from March 2017 to July 2019 in 9 public primary schools. The field study was conducted twice using the same cohorts first for baseline data collection and one year later for the follow-up data to accommodate the on-going mass administration of medicines by the Neglected Tropical Unit of Anambra State Ministry of Health in collaboration with the donor agency, Carter Centre.

This study focused specifically on primary school pupils, aligning with the Mass Administration of Medicines (MAM) programs implemented by Anambra State. These programs targeted primary school children due to the higher prevalence of helminth infections within this age group, as identified by earlier epidemiological assessments^2,3^. By concentrating on primary schools, the study aimed to optimize resource allocation and intervention effectiveness, providing valuable insights into factors influencing helminth prevalence. All methods were performed in accordance with the relevant guidelines and regulations.

## Study population and sample size

### Study participants and sampling

Within the 3 selected LGAs, public primary schools were chosen based on the following inclusion criteria:

- Size of the school’s population – large population preferred.
- Schools with pupils not dewormed in the last 3 months.

Based on the above criteria, 9 public primary schools, 3 from each selected LGAs were randomly selected from each LGA. Chosen by balloting^17^. These were Community, Egede and Udoka Primary Schools in Achalla; Akpunoji, Igwebuike and Ogbugbogu Primary Schools in Ezinifite and Central, Community and Development Primary Schools in Nsugbe. A scoping visit to meet and provide school teachers with the objectives and other detailed information on the study was done.

### Selection of children

The study population consisted of 1046 pupils from 5 to 15 years from the selected public primary schools whose parents/guardians gave their consent. The assent of the pupils was also obtained.

The sample size of 1046 was determined using the formula^18^:

$$\:n\:=\:\:\frac{N{Z}^{2}(1-P)}{{d}^{2}\left(N-1\right)+{Z}^{2}(P-1)}$$

Where:

Where,

*N* = Population size, *N* = 2,182 (based on the total number of pupils in the three communities selected as obtained from Anambra State Universal Basic Education Board (ASUBEB).

*Z* = Z statistic for a level of confidence, *Z* = 1.96,

*P* = Expected proportion (in the proportion of one, *P*= 0.58^19^.

d = Precision (error of estimate), d = 0.05.

Substituting,

$$\:n\:=\:\:\frac{2182\:\times\:{1.96}^{2}(1-0.58)}{{0.05}^{2}\left(2182-1\right)+{1.96}^{2}(0.58-1)}$$

$$\:n\:=\frac{3521}{3.839}\:=917.2$$

To account for a 75% response rate^20^ and a 25% non-response rate, an additional 14% of the sample size ( $\:\frac{14}{100}\times\:917.2$= 128.4+ 917.2 = 1,045.6) was calculated and included, resulting in a final sample size of 1046 participants to optimize precision and minimize potential withdrawals. Participant recruitment employed a stratified sampling approach^18^ with inclusion criteria including written parental/guardian consent, the pupil’s assent to participate and a minimum 3-year residency in the areas. Exclusion criteria included a nonresponsive study population, those unwilling to provide consent, and recent migrants or temporary residents of the area. Hence, a total of 1046 pupils aged 5–16 years from randomly selected public primary schools were enrolled for this study.

### Parasitological examination

To determine the prevalence and intensity of helminth infections among the study population, we collected stool and urine samples from each child over two consecutive days during both baseline and follow-up surveys. Each sample was carefully collected and labelled with the participant’s serial number to ensure accurate tracking and analysis. All samples were freshly collected within the premises of the selected schools, ensuring immediate processing and minimizing the risk of sample degradation.

### Urine collection and examination

Urine samples were collected between 10:00 AM and 12:00 PM, a time selected to optimize egg detection, as recommended by WHO guidelines^21^. Each pupil was provided with a sterile, labelled urine collection bottle and was instructed to provide a midstream urine sample of at least 10 ml, including the last few drops, which often contain the highest concentration of *Schistosoma haematobium* eggs. The collection was supervised by researchers and teachers to ensure compliance with protocols and to maintain the integrity of the samples.

Recognizing that menstruation could affect haematuria counts and potentially lead to false positive results^22^, we temporarily excluded menstruating females from these assessments. These individuals were marked and revisited once their menstrual periods had ended to collect accurate data. This system of regular communication allowed participants to inform the study team when their menstruation ceased, ensuring that all eligible participants could be included in the study.

Immediately after collection, urine samples were processed and examined on-site at the school premises. This on-site processing was essential due to the field study setting, ensuring that samples were analyzed promptly to maintain their integrity and prevent contamination or degradation. Samples were kept in sterile, airtight containers before analysis. Reagent strips (Urine-9 parameters, Accu-answer urinalysis laboratory test strips, Guilin Zhonghui Technology Co., Ltd., Guilin, China) were used to estimate the amount of blood present in the urine. For parasitological examination, 5 to 10 ml of urine was filtered through a 13 mm polycarbonate membrane filter (Sterlitech Corporation, NY, USA). The filter, which captured any *S. haematobium* eggs present, was then transferred to a clean, grease-free microscope slide. The slides were examined under a microscope at 40x magnification to identify and count *S. haematobium *ova. Eggs were identified based on their characteristic morphology: they are typically oval-shaped with a terminal spine^21^. Infection intensity was classified as light (1 to 49 eggs per 10 ml of urine) or heavy (more than 49 eggs per 10 ml of urine), following WHO guidelines^21^.

### Stool collection and examination

Each pupil was given a universal sample bottle equipped with a small spoon for stool collection. Pupils were instructed to defecate on a clean piece of paper and to use the spoon to collect a sample of the stool, which was then placed inside the sample bottle and securely covered. These samples were freshly collected and immediately processed within the school premises. Stool samples were examined using the modified quantitative Kato-Katz method, following the protocols established by Martin and Beaver^23^.

Within the school premises, duplicate thick smears were prepared on microscope slides using 41.7 mg templates. The slides were allowed to clear for 10 to 25 min before examination under a microscope. This timing allowed for optimal visualization of helminth eggs. The number of eggs for each helminth species was recorded and multiplied by a factor of 24 to calculate the number of eggs per gram (EPG) of stool. The EPG was further adjusted using correction factors as described by Nawalinski et al.^24^ to estimate the intensity of infection accurately. Identification of intestinal parasites, including *S. mansoni *and other soil-transmitted helminths, was conducted according to WHO guidelines^21^.

### Quality control

Field assistants received training in questionnaire administration and sample collection. Researchers ensured questionnaire completeness and adherence to recommended timeframes for urine and stool sample collection. All slides were initially examined by skilled laboratory technologists with expertise in parasitology. To ensure the accuracy and reliability of the results, a random 10% of the slides were re-examined by one of the laboratory technologists, who was blinded to the initial results and independently examined a subset of the slides. This double-checking process was implemented to verify the original egg count, maintain the integrity of the results, and minimize the risk of false positives. The results from both technologists’ examinations were then compared for consistency.

### Assessment of the efficacy of drug administration programs

To evaluate the effectiveness of the Mass Administration of Medicines (MAM) programs in reducing schistosomiasis and soil-transmitted helminthiasis among school-aged children, we followed this approach:

1. Baseline Data Collection: Stool and urine samples were collected from children before the MAM to establish the prevalence of infections, serving as a reference for post-treatment comparisons.
2. Mass Administration of Medicines: Praziquantel and albendazole were administered simultaneously to target both schistosomiasis and soil-transmitted helminthiasis, ensuring comprehensive treatment.
3. Follow-up Examinations: One-month post-treatment, follow-up stool and urine samples were collected from the same cohort of school-aged children to assess changes in helminth egg counts and determine the program’s impact on reducing helminth infections. All follow-up examinations were conducted within the one-year timeframe in the study locations chosen by the NTD Unit, Anambra State Ministry of Health, in collaboration with the Federal Ministry of Health (FMOH) and the donor agency (Carter Center), which provided the drugs for the mass treatment.
4. Directly Observed Treatment: Drugs were administered under the supervision of healthcare personnel to ensure accurate dosing. Praziquantel was dosed at 40 mg/kg based on each child’s weight, and albendazole was provided as a single tablet^25,26^. Tablets were given orally, with instructions to swallow them with water, ensuring compliance and ease of intake.
5. Homegrown School Feeding Program (HGSFP): Established in 2016, the HGSFP provides nutritious meals to children in all public primary schools in Anambra State. This initiative plays a crucial role in supporting the Mass Administration of Medicines (MAM) programs by ensuring that children receive a healthy meal before taking the medication. This not only helps to minimize potential side effects of the drugs but also enhances their effectiveness. Additionally, the HGSFP has significantly improved school enrollment and attendance, thereby increasing the number of children who can benefit from deworming treatments^5^.

Participants were observed for any adverse reactions post-administration, and they were also encouraged to report any side effects experienced. This monitoring helped to ensure the safety and well-being of the children and provided valuable information for future treatment protocols.

Egg Reduction Rate (ERR): The effectiveness of the treatment was assessed by calculating the ERR and comparing the number of helminth eggs in stool or urine samples before and after treatment^25,27,28^. ERR was determined using the formula^27,28^:

$$\:ERR\:=\frac{\text{P}\text{r}\text{e}-\text{t}\text{r}\text{e}\text{a}\text{t}\text{m}\text{e}\text{n}\text{t}\:\text{e}\text{g}\text{g}\:\text{C}\text{o}\text{u}\text{n}\text{t}\:-\text{P}\text{o}\text{s}\text{t}-\text{t}\text{r}\text{e}\text{a}\text{t}\text{m}\text{e}\text{n}\text{t}\:\text{e}\text{g}\text{g}\:\text{c}\text{o}\text{u}\text{n}\text{t}}{\text{P}\text{r}\text{e}-\text{t}\text{r}\text{e}\text{a}\text{t}\text{m}\text{e}\text{n}\text{t}\:\text{e}\text{g}\text{g}\:\text{C}\text{o}\text{u}\text{n}\text{t}}\:\times\:\frac{100}{1}$$

A higher ERR indicated a significant reduction in infection levels, reflecting successful treatment outcomes^27^.

### Overall assessment of risk factors and community peculiarities

For the overall assessment of risk factors associated with Schistosomiasis and STH infections and the evaluation of the distinct characteristics of communities, surveillance visits, oral interviews, gender- sensitive focus group discussions of 8, and pre-tested questionnaires were employed. These assessments particularly focused on socio-demographic characteristics and behavioural variables chosen for the study.

#### Development of strategies for community involvement

Oral interview, focus group discussions and pre-tested questionnaire were employed for the overall assessment of the factors that could be responsible for ensuring the complete involvement of the people in the management and sustainability on the administration of medicines and the school feeding programme. Focus group discussions which was also gender sensitive were conducted in groups of four for all the parents and teachers that volunteered. All discussions were made in local languages (Igbo language) and simplified to the best understanding of all the participants in the selected communities.

#### Data analysis

Data analysis was performed using Minitab 17 software. The intensity of infection was categorized following WHO recommendations^21^. Descriptive statistics, including frequencies (n) and proportions (%), were calculated. Prevalence and 95% confidence intervals were determined for gender and age groups. Chi-square (χ2) tests were used to assess the association between prevalence and socio-demographic characteristics and behavioural variables. Kruskal-Wallis test in Minitab 17 were employed for variations and comparisons of arithmetic means. Additionally, Odds Ratios (ORs) were calculated to assess associations between binary outcomes and predictor variables. Multinomial Logistic Regression was used to model relationships with multiple categorical outcomes. The level of significance was set at *p* < 0.05.

## Results

A total of 1046 pupils (523 males and 523 females) with a mean age of 9.25 ± 0.126 years had complete parasitological data and were also administered questionnaire and were included for all subsequent analyses (Table 1).

**Table 1:** Overall Prevalence (%) of Helminth infection (Schistosomiasis and Soil-transmitted Helminthiasis based on gender and age (Baseline) in the study community.

		Mono-infection					Co-infection	Total		
Gender	Number Examined	*Ascaris lumbricoides*	*Trichuris trichiura*	Hookworm	*Schistosoma haematobium*	*Schistosoma mansoni*	*Ascaris* and Hookworm	Overall Helminth Infection	OR for Overall Helminth Infection (95% CI)	*P* value
		NI (%)	NI (%)	NI (%)	NI (%)	NI (%)	NI (%)	NI (%)		
Male	523	38 (7.3)	1(0.2)	1(0.2)	4(1.0)	0(0.0)	0(0.0)	44(8.4)	1.172 (0.746–1.842)	0.490^NS^
Female*	523	31(6.0)	5(1.0)	0(0.0)	1(0.2)	0(0.0)	1(0.2)	38(7.3)	1.00	
**Total**	**1046**	**69(7.0)**	**6(1.0)**	**1(0.1)**	**5(0.5)**	**0(0.0)**	**1(0.1)**	**82(8.0)**		
Age (years)										
5–8	412	22(5.3)	1(0.2)	0(0.0)	1(0.2)	0(0.0)	1(0.47)	25(6)	3.153 (0.964–10.311)	0.058^NS^
9– 12	545	44(8.1)	5(1.0)	1(0.2)	4(1.0)	0(0.0)	0(0.0)	54(10)	1.852 (0.547–6.273)	0.322^NS^
13–16*	89	3(3.4)	0(0.0)	0(0.0)	0(0.0)	0(0.0)	0(0.0)	3(3.4)	1.00	
**Total**	**1046**	**69(7.0)**	**6(1.0)**	**1(0.1)**	**5(0.5)**	**0(0.0)**	**1(0.1)**	**82(8.0)**		
P-value		0.195	0.733		0.44			0.131		

*NI*: Number Infected.

At baseline, 8.4% of male pupils and 7.3% of female pupils were infected, with an OR of 1.172 (95% CI: 0.746–1.842, *p* = 0.490), showing no statistically significant difference in infection risk between genders. The highest infection rate was observed in pupils aged 9–12 years (10%), followed by those aged 5–8 years (6%) and 13–16 years (3.4%). Odds ratios were 3.153 (95% CI: 0.964–10.311, *p* = 0.058) for ages 5–8 and 1.852 (95% CI: 0.547–6.273, *p* = 0.322) for ages 9–12, with no significant differences between age groups (Table 1).

At follow-up/surveillance, a year later, results showed that the overall prevalence of helminth infection was 6% (65/1046) with 5.0% of male pupils and 7.0% of female pupils being infected, with an OR of 1.439 (95% CI: 0.865–2.394, *p* = 0.161). No significant differences were observed in the prevalence of helminth infection based on gender and age (*P* > 0.05) among the pupils in the study area. There was Co-endemicity of urogenital schistosomiasis and soil-transmitted helminthiasis among the pupils was also observed. The following helminth ova were identified respectively; *A. lumbricoides* 2% (20/1046) and *T. trichiura* 2.2% (23/1046) only while for *Schistosoma* spp., only *Schistosoma haematobium* ova 2% (20/1046) was seen. Co-infection of 0.2% (2/1046) between *A. lumbricoides* and *S. haematobium* was also observed (Table 2).

**Table 2:** Overall Prevalence (%) of Helminth infection (Schistosomiasis and Soil-transmitted Helminthiasis) based on gender and age (at Follow-up/surveillance) in the Study Community.

		Mono-infection					Co-infection	Total		
Gender	**Number Examined**	*Ascaris lumbricoides*	*Trichuris trichiura*	Hookworm	*Schistosoma haematobium*	*Schistosoma mansoni*	*Ascaris lumbricoides* and *S. haematobium*	Overall Helminth Infection	OR for Overall Helminth Infection (95% CI)	*P*-value
		NI (%)	NI (%)	NI (%)	NI (%)	NI (%)	NI (%)	NI (%)		
Male^*^	523	7(1.0)	8(2.0)	0(0.0)	12(2.0)	0(0.0)	0(0.0)	27(5.0)	1.00	0.161^NS^
Female	523	13(3.0)	15(3.0)	0(0.0)	8(2.0)	0(0.0)	2(0.4)	38(7.0)	1.439(0.865–2.394)	
**Total**	**1046**	**20(2.0)**	**23(2.2)**	**0(0.0)**	**20(2.0)**	**0(0.0)**	**2(0.2)**	**65(6.0)**		
Age (years)										
5–8	412	7(2.0)	13(3.0)	0(0.0)	6(2.0)	0(0.0)	1(0.2)	27(7.0)	6.171 (0.827 −6.028)	0.076^NS^
9– 12	545	13(2.0)	10(2.0)	0(0.0)	13(2.0)	0(0.0)	1(0.2)	37(7.0)	6.409 (0.868–47.318)	0.069^NS^
13–16*	89	0(0.0)	0(0.0)	0(0.0)	1(1.0)	0(0.0)	0(0.0)	1(1.0)	1.00	
**Total**	**1046**	**20(2.0)**	**23(2.2)**	**0(0.0)**	**20(2.0)**	**0(0.0)**	**2(0.2)**	**65(6.0)**		
P-value		0.18			0.5			0.14		

*NI*: Number Infected.

Although there was a decline in the overall prevalence of helminth infection by 2%, there was an increase in the total number of infected pupils with *S. haematobium* by 1% (15/1046) while a decline in the number of infected pupils with STHs by 3% (32/1046) was seen.

Result showed that the overall prevalence of Helminth infection (Schistosomiasis and Soil-transmitted helminthiasis) varied significantly at baseline (*P* = 0.00) and follow-up (*P* = 0.01) respectively with respect to location (Table 3).

**Table 3:** Overall Prevalence (%) of Helminth infection ((Schistosomiasis and Soil-transmitted Helminthiasis) with respect to Location (at baseline and Follow-up/surveillance) in the Study Community.

Location		Mono-infection					Co-infection	Total		
**Baseline**	**Number Examined**	***Ascaris lumbricoides***	***Trichuris trichiura***	**Hookworm**	***Schistosoma haematobium***	***Schistosoma mansoni***	*Ascaris lumbricoides * **and Hookworm**	**Overall Helminth Infection**	**OR for Overall Helminth Infection** **(95% CI)**	**P-value**
		**NI (%)**	**NI (%)**	**NI (%)**	**NI (%)**	**NI (%)**	**NI (%)**	**NI (%)**		
Achalla	390	57(15.0)	6(2.0)	1(0.3)	3(1.0)	0(0.0)	1(0.3)	68(17.4)	3.423(1.062–11.028)	0.04 ^*S*^
Ezinifite*	375	3(1.0)	0(0.0)	0(0.0)	1(0.3)	0(0.0)	0(0.0)	4(1.1)	1.00	
Nsugbe	281	9(3.0)	0(0.0)	0(0.0)	1(0.4)	0(0.0)	0(0.0)	10(4.0)	19.587 (7.067–54.285)	0.00 ^*S*^
**Total**	**1046**	**69(7.0)**	**6(1.0)**	**1(0.1)**	**5(0.5)**	**0(0.0)**	**1(0.1)**	**82(8.0)**		
P-value		0.00 ^*S*^	0.01 ^*S*^		0.57			0.00 ^*S*^		
Follow-up							*Ascaris* and *S. haematobium*			
Achalla	390	13(3.3)	10(2.6)	0(0.0)	11(2.8)	0(0.0)	1(0.3)	35(9.0)	2.070(0.980–4372)	0.06^NS^
Ezinifite*	375	3(1.0)	6(1.6)	0(0.0)	3(0.8)	0(0.0)	0(0.0)	12(3.0)	1.00	
Nsugbe	281	4(1.4)	7(2.5)	0(0.0)	6(2.1)	0(0.0)	1(0.4)	18(6.0)	2.982(1.523–5.839)	0.00 ^*S*^
**Total**	**1046**	**20(2.0)**	**23(2.2)**	**0(0.0)**	**20(2.0)**	**0(0.0)**	**2(0.2)**	**65(6.0)**		
P-value		0.034 ^*S*^	0.63		0.12			0.01 ^*S*^	0.01 ^*S*^	

*NI*: Number Infected.

At baseline, the overall helminth infection prevalence varied significantly across locations. Achalla recorded the highest prevalence (17.4%), followed by Nsugbe (4.0%) and Ezinifite (1.1%). Achalla showed significantly higher odds of infection compared to Ezinifite (OR: 3.423; 95% CI: 1.062–11.028; *p* = 0.04), while Nsugbe exhibited the highest odds (OR: 19.587; 95% CI: 7.067–54.285; *p* = 0.00). At follow-up, overall prevalence declined across all locations. Achalla reduced to 9.0%, Nsugbe to 6.0%, and Ezinifite remained low at 3.0%. Despite the reduction, Nsugbe continued to show significantly higher odds of infection compared to Ezinifite (OR: 2.982; 95% CI: 1.523–5.839; *p* = 0.00) (Table 3).

For species-specific prevalence, Achalla consistently recorded the highest rates at baseline. *Ascaris lumbricoides* prevalence was 15% (57/390) and *Trichuris trichiura* prevalence was 2% (6/390), both significantly higher than in other locations (*p* = 0.00 and *p* = 0.01, respectively). Similarly, *Schistosoma haematobium* prevalence was highest in Achalla (1%; 3/390), compared to Nsugbe (0.4%; 1/281) and Ezinifite (0.3%; 1/375). Significant differences in *Ascaris lumbricoides* prevalence were observed across locations at both baseline (*p* = 0.00) and follow-up (*p* = 0.034) (Table 3).

The prevalence of species-specific *Schistosoma* infection showed that individual infections were generally low (< 5%) with only *S. haematobium* found both at baseline and follow-up study respectively. At baseline, *S. haematobium* infection intensities were primarily of heavy infection except in a pupil with a light infection intensity. At Follow-up, *S. haematobium* infection intensities were mainly of light infection except in 7 pupils with heavy intensity of infection. (Table 4).

**Table 4:** Overall prevalence and intensity of *Schistosoma haematobium* infection among the Study population at Baseline and Follow up in the study area.

Total sample (*N* = 1046)	Overall prevalence	Mean interval (x̄ ± σ)	EPG Range	95% Confidence Interval (CI)	Light intensity (%)	Heavy intensity (%)
At Baseline						
*Schistosoma haematobium*	5(0.5)	38.39 ± 14.428	24–53	(37.52–39.26)	1(20)	4(80)
At Follow-up						
*Schistosoma haematobium*	20(2.0)	47.83 ± 9.856	38–58	(47.23–48.43)	13(65)	7(35)

This study also confirmed the evidence of female genital schistosomiasis (FGS) seen in a female pupil that had a heavy intensity of infection and this was published^29^.

At baseline, there was moderate intensity of infection with *A. lumbricoides* only among the pupils in Achalla. The intensity of parasite load amongst children who were positive for *T. trichiura* and Hookworm was of light intensity at baseline for the three communities studied. Follow-up study showed that there was light intensity of infection for all STHs infection seen in all the locations studied (Table 5).

**Table 5:** The Intensity of Soil-transmitted Helminth infection among the Study Population at Baseline and Follow-up in the Study Community.

Location of Primary schools	Survey phases	STHs infection	Mean interval (x̄ ± σ)	EPG Range	95% CI	Light intensity (%)	Moderate intensity (%)	Heavy intensity (%)	*P*-value
Achalla	Baseline	*A. lumbricoides*	5001.97 ± 2215.778	1000–8000	(4600.45–5403.49)	52	5	0	
		*T. trichiura*	279.64 ± 234.301	50–500	(250.71–308.57)	6	0	0	
		Hookworm	45 ± 63.64	10–100	(39.00–51.00)	1	0	0	
	Follow-up	*A. lumbricoides*	207.95 ± 437.101	50–500	(150.00–265.00)	13	0	0	
		*T. trichiura*	257.81 ± 225.537	100–400	(230.60–285.02)	10	0	0	
		Hookworm	0.00 ± 0.00	0	0	0	0	0	
									0.00 ^*S*^
Ezinifite	Baseline	*A. lumbricoides*	930 ± 936.75	300–1500	(800.00–1060.00)	3	0	0	
		*T. trichiura*	0.00 ± 0.00	0	0	0	0	0	
		Hookworm	0.00 ± 0.00	0	0	0	0	0	
	Follow-up	*A. lumbricoides*	413.33 ± 225.462	200–600	(380.00–446.66)	3	0	0	
		*T. trichiura*	190.42 ± 91.93	100–300	(175.00–205.84)	6	0	0	
		Hookworm	0.00 ± 0.00	0	0	0	0	0	
Nsugbe	Baseline	*A. lumbricoides*	508.06 ± 753.268	200–1000	(420.00–596.12)	9	0	0	
		*T. trichiura*	0.00 ± 0.00	0	0	0	0	0	
		Hookworm	0	0	0	0	0	0	
	Follow-up	*A. lumbricoides*	4	50–200	(105.00–117.50)	7	0	0	
		*T. trichiura*	7	50–250	(155.00–179.28)	0	0	0	
		Hookworm	0	0	0	0	0	0	

**Fig. 2:**
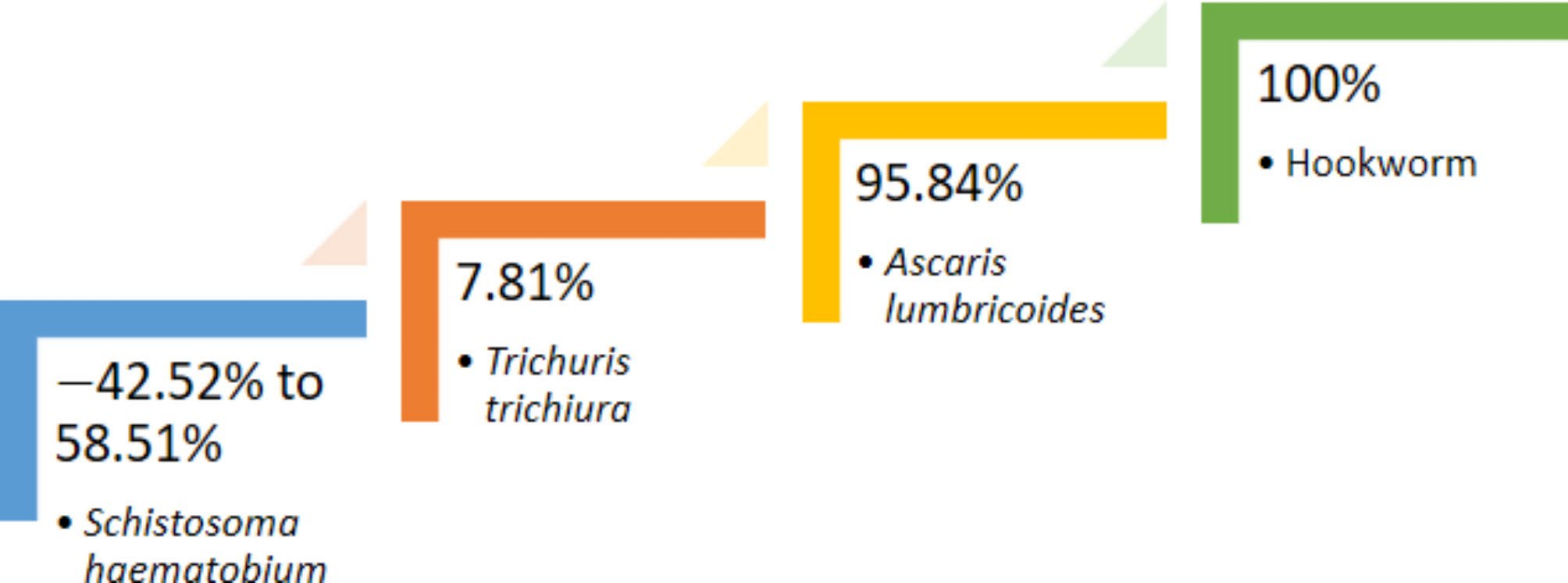
Egg Reduction Rate (ERR) of identified helminths among the Study Population.

For *S. haematobium*, the pre-treatment egg count ranged from 23.962 to 52.818 eggs, with a mean of 38.39 eggs (± 14.4280). Following treatment, the post-treatment egg count ranged from 37.974 to 57.686 eggs, with a mean of 47.83 eggs (± 9.856). This resulted in an ERR that ranged from − 42.52 to 58.51%. For STH infection, the results revealed a high ERR for *A. lumbricoides* (95.84%), indicating a significant reduction in egg count after treatment. For *T. trichiura*, the ERR was comparatively lower (7.81%), suggesting a lesser reduction in egg count. Remarkably, a 100% ERR was observed for Hookworm, signifying the elimination of detectable eggs post-treatment (Fig. 2).

**Fig. 3:**
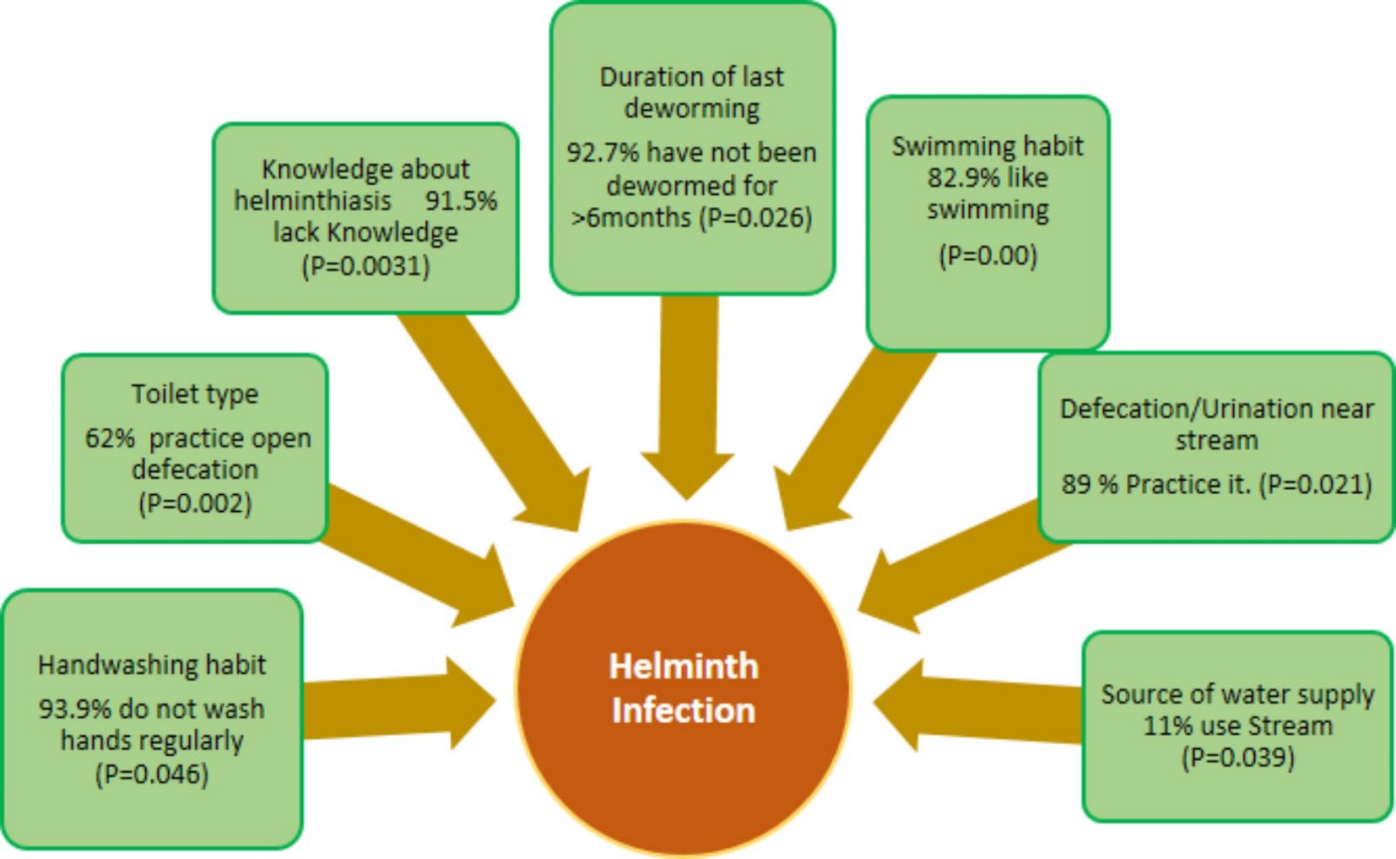
Risk factors associated with Urogenital Schistosomiasis and Soil-transmitted helminthiasis among the Study Population in the study community.

**Fig. 4:**
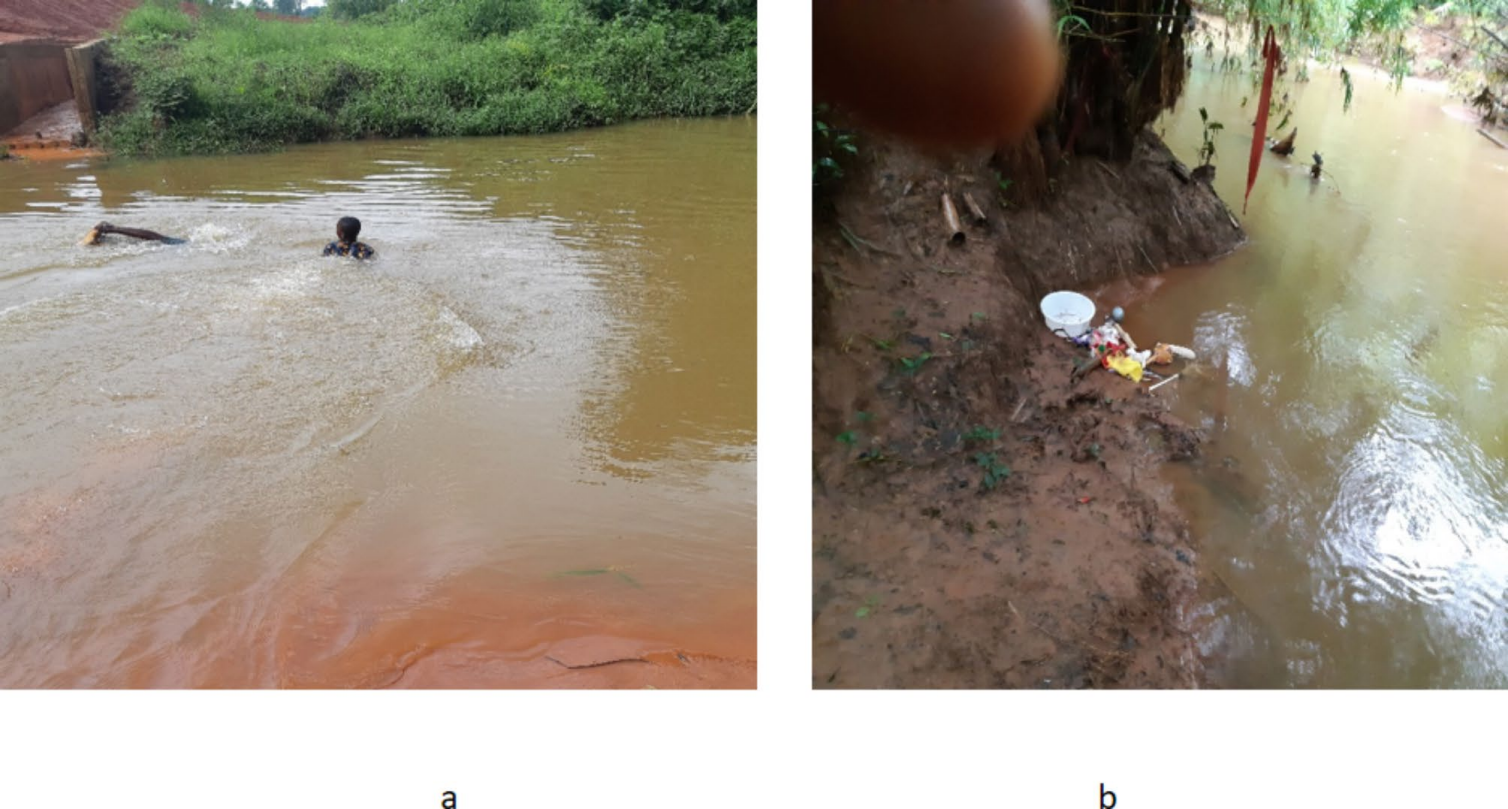
Water contact activities (**a**) Children swimming in the river (**b**) Fetish items placed by traditional worshippers.

**Fig. 5:**
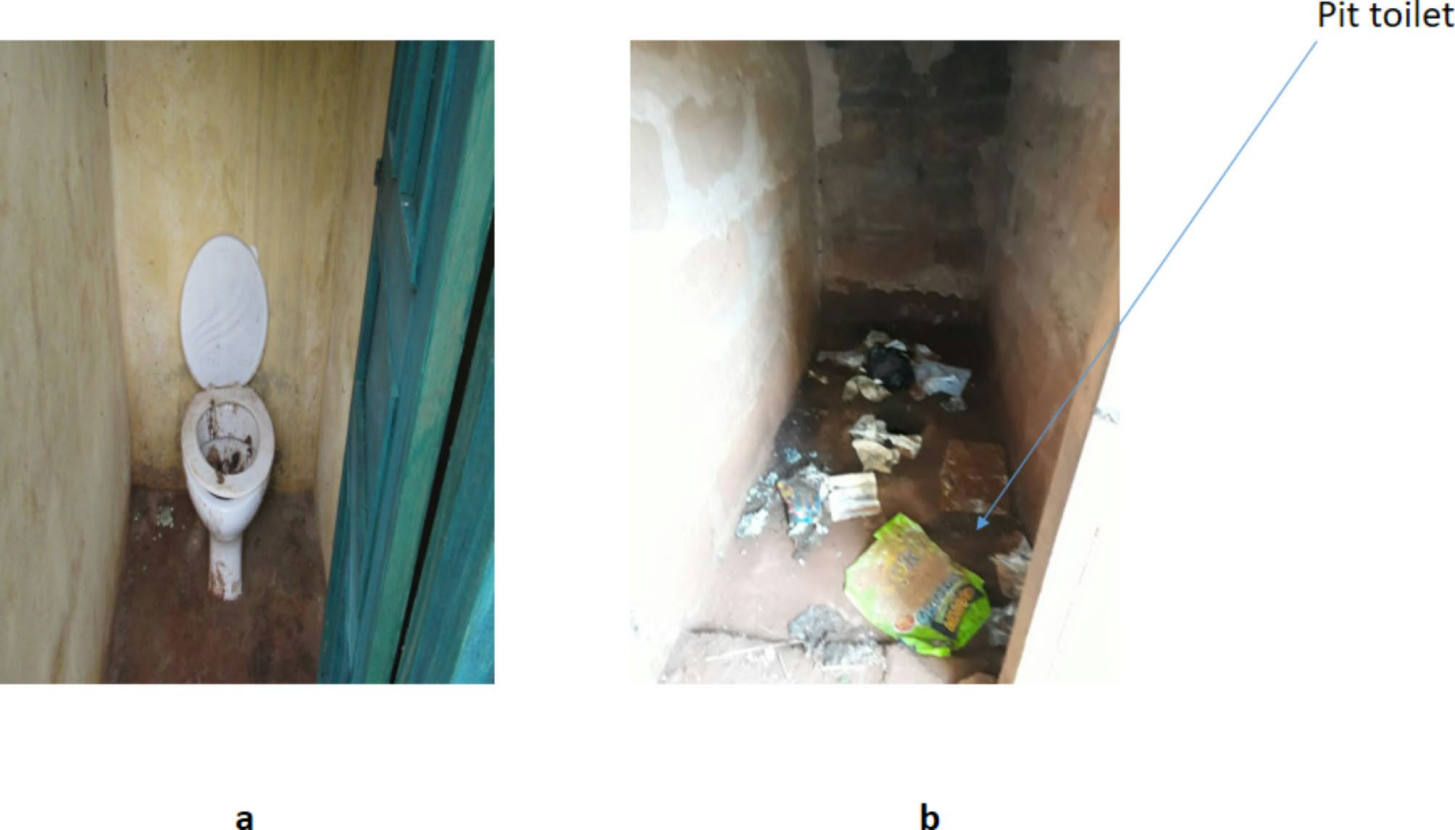
Poor toilet facilities (**a**) and (**b**): Dirty toilets seen in some of the schools.

**Fig. 6:**
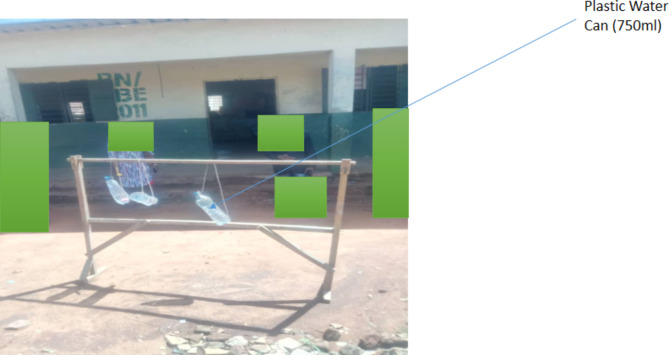
Makeshift hand washing facility used in the school because of lack of water supply.

The assessment of the risk factors associated with Urogenital Schistosomiasis and Soil-Transmitted Helminth (STH) infections revealed significant socio-demographic and behavioral variables among the study population. Results showed that among the 1046 pupils examined, 8% (82/1046) were found to be infected with helminthiasis. Among the infected pupils, 93.9% (77/82) do not wash hands regularly, 91.5% (75/82) lack knowledge about helminthiasis transmission, 62% (51/82) practice open defecation in the bush, 92.7% (76/82) have not been dewormed for more than 6 months, 82.9% (68/82) have a preference for swimming, 11% (9/82) use a stream as their primary water source, and 89% (73/82) practice defecation/urination near the stream. The study found that hand washing habits (*P* = 0.046), knowledge about helminthiasis (*P* = 0.0031), type of toilet used (*P* = 0.002), duration since last deworming (*P* = 0.026), swimming habits (*P* = 0.00), source of water supply (*P* = 0.039), and defecation/urination near streams (*P* = 0.021) were significantly associated with the prevalence of these infections (Fig. 3). Furthermore, observations (Figs. 4, 5 and 6) provided additional context to these findings.

Assessment of the best ways to ensure complete community involvement in the management and sustainability of medicine administration for the control and possible elimination of schistosomiasis and soil-transmitted helminthiasis revealed the following: Among the 243 respondents, 87% earn less than $50 per month, 39% practice open defecation, and 71% advocated for the continuation of Mass Administration of Medicines, with 67% appreciating that it was free of charge. Regarding sustainability, 98% were satisfied that the government is providing funds (Table 6).

**Table 6:** Assessment of Socio-demographic characteristics and perceptions of community members about the benefits of school feeding and mass administration of medicines in the study area.

Total number of Respondents (*N* = 243)		(%)
Occupation		
	Teacher	20 (2.4)
	Farmer	100 (41.2)
	Trader	55(22.6)
	Civil servant	8 (75)
	Health worker	15(6.2)
	Food vendor	45 (18.5)
Estimate of money earned in a month		
	Less than ₦11,500 ($25)	23(9.5)
	Less than or equal to ₦23,000 ($50)	189 (78)
	More than ₦23,000 ($50)	31(13)
Type of Toilet Facility at home		
	Pit	37(15.2)
	Water Closet	111(46)
	Bush	95 (39.1)
Source of water used at home		
	Bore hole/Tap water	131(54)
	Rain water	29(12)
	Stream	83(34)
Teaches the child personal hygiene		
	Always	101(42)
	Sometimes	57 (23.5)
	Not applicable	85 (35)
Frequent inspection of the child’s fingernails		
	Yes	52 (21)
	No	113(47)
	Seldom	78(32)
Time of last deworming of the children/Household		
	1–5 months	37(15.2)
	6–11 months	151(62)
	1 year and above	55(22.6)
Does your Child like the Home-grown School feeding in his/her School		
	Yes	232(96)
	No	11(4.53)
Number of times your child eats at home during School days		
	Once	89(37)
	Twice	107(44)
	Thrice	47(19)
Does the Home-grown School feeding substitute the child’s ration at home?		
	Yes	196(81)
	No	47(19)
When your child is ill, how do you treat the child?		
	Hospital	10(4)
	Herbalist	92(38)
	Chemist	141(58)
When last did you deworm yourself?		
	1–5 months	8(3)
	6–11 months	201(83)
	1 year and above	34 (14)
Do you like the mass drug administration of medicines?		
	Yes	172(71)
	No	71(29.2)
If yes, why do you like it?		
	Effective	57(33)
	Free	115(67)
How best do you think the programme can be sustained?		
	Funding by Government	183(75)
	Funding by individuals	60 (25)
If you are asked to contribute some funds to sustain the programme, will you like it?		
	Yes	6(3)
	No	237(97)
How best do you think drugs can be properly distributed?		
	By use of Community drug distributors (CDDs)/Community Health Extension Workers (CHEWS)	225(93)
	Primary Health Centers	18(7)
What gender of CDDs/CHEWS do you prefer?		
	Male	91(37)
	Female	145(60)
	Any	7(3)
Will the gender of CDDs/CHEWS affect your decision to take the drugs		
	Yes	179(73.7)
	No	64(26.3)
If yes, why do you prefer a particular gender?		
	Cultural belief	105(58.7)
	Trust	51(28.5)
	Commitment	21(11.7)
	Indecisive	2 (1.1)

Furthermore, Oral interview and Focus group discussions provided additional context to these findings. For example,

One respondent who sold food at the local market shared,

***“I am the breadwinner of the family and any money I get goes to feeding and paying their school fees. I cannot afford to give my children ‘ogwu okpo’ (anthelminthic drugs) every three or six months. Where will I get the money?’’ (Female Respondent A)***.

Another respondent said,

***“Who has time to take drugs when you are not sick? I only take the medicines when the government shares them and I keep them until anyone falls sick***,*** then I give the medicines to them.’’(Male Respondent A)***.

Another respondent a local CHEW had this to say,

***“We need more awareness of the importance of regular deworming. Many of us in the community are unaware of or neglect it.’’ (Female Respondent B)***.

A concerning 39% reported open defecation practices.

One respondent gave the following reason,

***“We do not have water***,*** we have to buy or fetch from the stream. It is easier to go to the bush or farmlands***,*** that way***,*** we save more water.” (Female Respondent C)***.

Another respondent, a teacher also echoed the lack of water as a reason for open defecation, adding that,

***“Even the school lacks water***,*** and the pupils have to go a reasonable distance just to get water. The toilet facilities are very poor too. So what do you expect us to do? We know that open defecation is bad***,*** and hygiene is important***,*** but all the initiatives we have had so far have fallen short because there is no water.” (Female Respondent D)***.

The majority (71%) of respondents favoured Mass Administration of Medicines (MAM), primarily due to its free administration.

A respondent reported,

***“I like that the drugs are free. For most of us***,*** these free drugs are the health care we can afford. It is a good thing.” (Male Respondent B)***.

In contrast, one respondent said,

***“I don’t trust these drugs. Most of them might be expired or something worse. I prefer to go to the chemist and buy exactly what I want. I think it is safer.” (Male Respondent C)***.

A majority of the respondents, 81% believed that school feeding programs substituted their child’s ration at home.

A respondent reported,

***“The food my children are given in school helps a lot. I don’t have to worry about feeding them when they get back from school.” (Female Respondent E)***.

Another respondent had this to say,

***“I like the school feeding oo. But I will still feed my children when they get back. I think the school feeding has added to what they get. My children look plumper and fresher. I like it.” (Female Respondent F)***.

While 75% suggested that the government should fund MAM for sustainability, 98% objected to contributing funds themselves.

In this regard a vocal respondent shared,

***“If we had the money***,*** we would be buying these drugs ourselves. Already we are struggling with a lack of many necessary things even roads. These free drugs are the least of what the government owes us.” (Male Respondent D)***.

Respondents recommended the use of Community Drug Distributors (CDDs)/Community Health Extension Workers (CHEWs) for drug administration, with a preference for female CDDs/CHEWs (60%).

One respondent shared,

***“I like to see females who we know sharing these drugs (CHEWs). We trust them more.” (Male Respondent E)***.

Concerning preference, one respondent shared,

***“I don’t know why***,*** but I just like to be treated by the female nurses. They are also gentler with children. And when they come to your house***,*** you don’t have to worry that you are in some sort of trouble.” (Female Respondent G)***.

Regarding child treatment practices, 58% relied on chemists, 38% visited herbalists, and only 4% sought treatment at hospitals.

One respondent shared,

***“Using herbs or going to the chemist to mix drugs is cheaper. Hospital treatments are very costly and health centres are not always open***,*** because they do not have drugs.” (Male Respondent F)***.

## Discussion

This study confirmed that helminth infections remain prevalent in communities in Anambra State despite ongoing Mass Administration of Medicines (MAM) efforts. Among the primary school pupils sampled, the overall prevalence of helminth infection was relatively low at 8% at baseline and 6% during follow-up, which aligns with similar studies across sub-Saharan Africa that have reported reductions in infection rates following MAM initiatives^30,31^. Our findings reveal that although MAM has reduced the overall burden of infection, several underlying factors contribute to reinfection, making it clear that MAM alone is insufficient for sustainable control. The persistence of helminths highlights the need for a more integrated approach that goes beyond medication distribution to address environmental, behavioural, and socio-cultural factors.

Key contributors to the continued presence of helminths include inadequate water and sanitation infrastructure, lack of comprehensive health education, and socio-cultural barriers to drug uptake. Many communities, particularly those in peri-urban and rural areas, still struggle with poor access to clean water and proper sanitation, conditions that facilitate the transmission of helminths. The persistence of open defecation, mentioned by 39% of respondents as a necessity due to lack of water, highlights critical gaps in WASH (Water, Sanitation, and Hygiene) infrastructure that undermine sustainable NTD control efforts. Without addressing these foundational WASH issues, achieving the WHO 2030 Road Map goals for NTD elimination will remain a significant challenge. Additionally, the low levels of health literacy and persistent cultural beliefs about medicine further impede the success of MAM programs, as many people remain hesitant to participate fully in these campaigns. This calls for more robust, culturally sensitive community engagement to foster trust and increase compliance. This issue mirrors findings from Kenyan research, where community-specific challenges have contributed to the persistence of helminth infections despite regular MAM^32^. This shows that there is a need to understand contextual cultural values as this has a great influence on the uptake of any health intervention in the communities^29,33^. This will help to determine the best culturally congruent approach to the provision of healthcare in these endemic communities that will be implemented. This study therefore emphasizes the importance of socio-cultural factors, such as mistrust in health programs and misinformation, which have been less explored in previous studies. Addressing these socio-cultural issues is crucial for improving community participation and compliance.

A notable challenge to MAM effectiveness identified in this study was compliance, with some children initially pretending to take medication but subsequently spitting it out. This issue was mitigated by implementing Directly Observed Therapy (DOT) and reinforcing the importance of adherence through education, improving compliance, and aligning with successful Ugandan studies that applied similar behavioural interventions^34^. Such adherence strategies are crucial for maximizing MAM impact and reducing helminth transmission.

Additionally, while MAM campaigns have achieved measurable success in reducing infection rates, the study found that reinfection remains a significant issue, especially in peri-urban areas where one might expect lower transmission due to better infrastructure. This finding challenges assumptions that urbanization alone leads to better health outcomes and suggests that localized factors, such as intermittent access to sanitation and unregulated water sources, may play a larger role in these communities than previously acknowledged.

Co-endemicity of urogenital schistosomiasis and soil-transmitted helminthiasis was observed, albeit with a low co-infection rate of 0.2% for *A. lumbricoides* and *S. haematobium*. Although low, this co-infection rate suggests a need for integrated control programs to address overlapping environmental and behavioural factors contributing to these infections^5,35,36^. Combined interventions are more effective for co-endemic areas^37,38^. Targeted preventive chemotherapy in such areas is essential to mitigate potential health impacts associated with co-infections. Encouragingly, a declining trend in helminth prevalence was observed throughout this study, consistent with recent studies^5,39^, likely due to the ongoing MDA efforts facilitated by organizations like the Carter Center in partnership with federal and state health ministries. However, *A. lumbricoides*prevalence remained notably higher than other STHs at both baseline (7%) and follow-up (2%), a pattern consistent with other studies^5,36^ that identify this parasite as particularly resilient. This persistence suggests a need for targeted interventions to specifically address *A. lumbricoides*, which may respond less effectively to standard MAM protocols.

Concerningly, an increase in the prevalence of *Schistosoma haematobium*was observed during the follow-up, likely due to the lack of MAM coverage in non-schisto-endemic areas. Untreated infections in even a single individual can perpetuate transmission within a community, underscoring the need for comprehensive mapping and inclusion of all at-risk areas to prevent untreated communities from acting as reservoirs for infection^40,41^. Expanding MAM to all communities with potential exposure could significantly reduce the risk of transmission and reinforce the progress made by MAM efforts.

Finally, this study was limited to primary school pupils, as MAM programs in Anambra State specifically target this high-risk age group. Secondary school students were excluded to streamline resource management and intervention delivery. Expanding future studies to include older age groups, such as secondary school students, would allow for a broader understanding of helminth infection dynamics and provide insights for more comprehensive public health strategies that build on these findings. This could support more inclusive, sustainable interventions that contribute to lasting reductions in infection rates and align with global neglected tropical disease (NTD) elimination goals.

The longitudinal nature of the study is a key strength, as it allowed for the tracking of infection rates and community responses to MAM over an extended period, providing a more in-depth understanding of the factors at play than a cross-sectional approach would allow. Additionally, the study’s large sample size and its representation of diverse communities across Anambra State strengthen the generalizability of the findings. However, the study’s limitation to a single state means that broader regional patterns remain unexplored, and future research should expand to different geographic areas in Nigeria or other sub-Saharan African countries to validate these findings.

The practical implications of this study are significant. The persistence of helminth infections suggests that policymakers must adopt a more holistic strategy. While MAM remains a vital tool, it should be integrated with WASH initiatives, community health education, and tailored local interventions to reduce environmental risk factors and improve drug uptake. Additionally, involving local leaders and health workers in education campaigns could help dispel myths and encourage wider participation in future MAM rounds. Further, implementing community-based surveillance systems could enable health authorities to monitor infection and reinfection rates more effectively, allowing for adaptive interventions based on real-time data.

In conclusion, this study contributes to the broader discourse on helminth control by highlighting the limitations of MAM as a standalone intervention and advocating for a more integrated approach. The findings suggest that health policies must focus on environmental, socio-cultural, and behavioural factors in addition to medical interventions to achieve sustainable reductions in helminth prevalence. Future research should consider broader population groups, such as secondary school students, to better understand infection dynamics and inform more comprehensive public health strategies. The study consequently advocates the need for the government to introduce vocational programs and improve basic social amenities in these communities, as these efforts will help to improve the financial status and overall well-being of community members. By addressing both the health and socioeconomic needs of affected populations, these strategies hold promise for enhancing the long-term effectiveness and sustainability of helminth control efforts in Anambra State and similar settings.

## Data Availability

All relevant data supporting the findings of this study are included in the manuscript.
